# *Anaplasma* species of veterinary importance in Japan

**DOI:** 10.14202/vetworld.2016.1190-1196

**Published:** 2016-11-04

**Authors:** Adrian Patalinghug Ybañez, Hisashi Inokuma

**Affiliations:** 1 Biology and Environmental Studies Program, Sciences Cluster, University of the Philippines Cebu, Lahug, Cebu City 6000, Philippines; 2 Department of Veterinary Clinical Science, Obihiro University of Agriculture and Veterinary Medicine, Obihiro, Inada Cho, Hokkaido 080-8555, Japan

**Keywords:** *Anaplasma* spp, Japan, tick-borne pathogen

## Abstract

*Anaplasma* species of the family Anaplasmataceae, order Rickettsiales are tick-borne organisms that can cause disease in animals and humans. In Japan, all recognized species of *Anaplasma* (except for *Anaplasma ovis*) and a potentially novel *Anaplasma* sp. closely related to *Anaplasma phagocytophilum* have been reported. Most of these detected tick-borne pathogens are believed to be lowly pathogenic in animals in Japan although the zoonotic *A. phagocytophilum* has recently been reported to cause clinical signs in a dog and in humans. This review documents the studies and reports about *Anaplasma* spp. in Japan.

## Introduction

**Figure-1:**
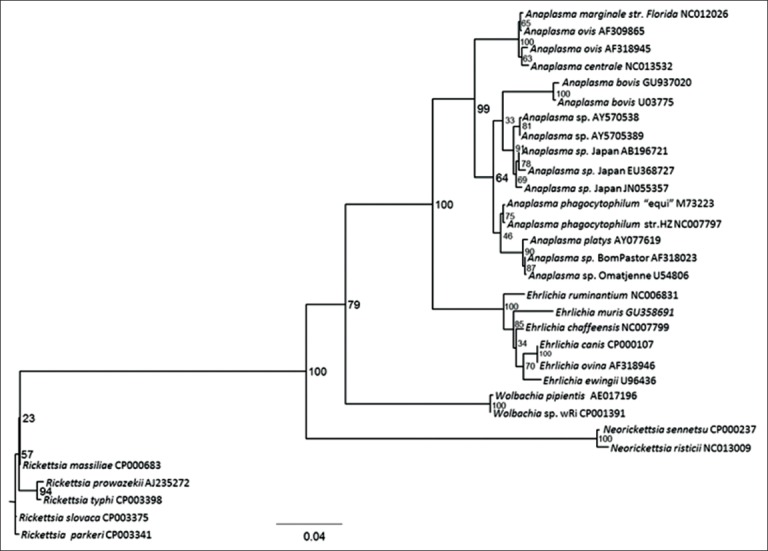
Phylogenetic tree of *Anaplasma* spp. based on the 16S rRNA gene using maximum likelihood. *Rickettsia parkeri* was set as the out group [3].

*Anaplasma* species are Gram-negative, obligate intracellular bacteria of the order Rickettsiales, family Anaplasmataceae. These bacteria are transmitted by ticks. Currently, there are six recognized species under this genus: *Anaplasma ovis*, *Anaplasma marginale*, *Anaplasma centrale*, *Anaplasma platys*, *Anaplasma bovis*, and *Anaplasma phagocytophilum* (Figure-1) [1-3]. All of these species have been reported in Japan except for *A. ovis*, which is an intraerythrocytic bacteria that may infect goats, sheep [4], and cattle [5]. *A. ovis* has been reported in wildlife, including reindeer (*Rangifer tarandus*) [6] and European roe deer (*Capreolus* *capreolus*) [3] in other countries. As Japan has an increasing deer population, it may be worthwhile to attempt to specifically detect this pathogen because it can be pathogenic in the deer species [5,6]. On the other hand, recent reports on *Anaplasma* or *Ehrlichia* spp. infections in dogs in Japan remained low (1.5%) [7], similar to previous findings (1.1%) of Sakamoto *et al*. [8] but lower than the findings (7.5%) of Inokuma *et al*. [9].

Results from previous studies have suggested the presence of a potentially novel *Anaplasma* sp. closely related to *A. phagocytophilum* in Japan (herein referred to as *Anaplasma* sp. Japan) due to the low 16S rRNA gene identities and phylogenetic divergence of the detected bacterium with registered *A. phagocytophilum* sequences [10-15]. Phylogenetic inferences have suggested that 2 clades exist within the genus *Anaplasma*: (1) Erythrocytic (*A. marginale, A. ovis*, *A. centrale*) and (2) leukocytic (*A. bovis, A. phagocytophilum, A. platys, Anaplasma* sp. Japan) [3]. In this review, studies on *Anaplasma* species detected in Japan are briefly summarized.

### A. marginale

*A. marginale*, the most common etiologic agent of bovine anaplasmosis, is endemic worldwide especially in tropical and subtropical areas [16]. This pathogen can be detected by peripheral blood smear and molecular means [17,18] and can be genetically diverse [19]. It mainly affects cattle [20,21], causing mild to severe febrile hemolytic anemia that can result in considerable economic losses to both dairy and beef industries [1,22]. It is transmitted biologically by ticks (e.g.: *Dermacentor andersoni*, *Rhipicephalus (Boophilus) microplus*) [1] although it can also be transmitted mechanically by blood-contaminated mouthparts of biting flies and mosquitoes [23]. Erythrocytes are the major sites of infection in cattle [22], in which *A. marginale* may be seen near the margins of the infected cell. *A. marginale* can be transmitted transplacentally from dam to calves [24]. *A. marginale* was previously distributed only in the Okinawa Prefecture [25] although an outbreak was reported in imported cattle in Tokyo in 1989 [26]. Detection of *A. marginale* DNA fragments in Japan was last reported in a Japanese black cow in Okinawa in 2009, which was 13 years after eradication of *R. (Boophilus) microplus* [25]. It was believed that the cow was persistently infected after it was first exposed when it was still a calf. Since then, there have been no detection reports of the pathogen in Japan. It is apparent that are no more viable tick vectors that can transmit this pathogen in the country. Recent studies on ticks present in the country reported several species which are not known to be capable of transmitting *A. marginale* [27].

The protein composition and epitope sites of *A. marginale* in Japan are already analyzed [28,29]. It has also been molecularly characterized based on the major surface protein 1 alpha (MSP1a) wherein 4 tandem repeats that are unique to the Japanese strain were found [25]. Using the 16S rRNA and heat shock operon (*groEL*) genes [25], DNA fragments of Japanese *A. marginale* were found 100% identical to Taiwanese (JQ321376), Chinese (DQ341369), American (CP000030, AF311303, AF309866) and Australian (AF414874, CP006846-7) strains, and 99.9% identical with Australian (CP006846-7, AF414860) and Israel (AF414861-2), respectively. Due to high similarities, it was expected that Japanese *A. marginale* strain would group together with the strains from other countries in the phylogenetic analyses using the two genes.

While several countries have progressed in the development of detection tools and vaccines against *A. marginale*, recent researches about this pathogen in Japan have been limited, specifically detection methods using the heat shock operon (*groESL*) and MSP5 genes [17,21].

### A. platys

*A. platys* is known to infect platelets in dogs [30], causing a disease called canine cyclic thrombocytopenia. It is believed to be transmitted by the brown dog tick Rhipicephalus sanguineus [31,32]. Brown dog ticks (R. sanguineus) and dogs that were seropositive and/or PCR positive with A. platys have been reported in Fukushima, Miyazaki, Kagoshima, Ishigaki, Yamaguchi and Okinawa, and Japan [9,31,33-36]. Although observation of A. platys inclusion bodies in the peripheral blood platelets was reported [9], positive dogs did not show any obvious clinical sign that was suggestive of the infection [37]. To date, there are no clinical reports suggesting active clinical infection caused by this pathogen in dogs in Japan.

### A. centrale

*A. centrale* is an intraerythrocytic tick-borne rickettsia of cattle that has a different morphology and virulence compared to *A. marginale*. Although severe disease may also occur with *A. centrale*, it can cause asymptomatic or paucisymptomatic infections [22], or only a mild anemia in most cases [1,34]. *A. centrale* is used for extensive vaccination of cattle against *A. marginale* infection in endemic areas [22]. Using microscopy, *A. centrale* inclusions appear centrally located [38]. Similar to *A. marginale*, there have been no recent detection reports of *A. centrale* in Japan, except in wild deer and *Haemaphysalis longicornis* ticks [39,40]. Sequence identities of the Japanese *A. centrale* (Aomori) have been shown to have the lowest similarities with other *A. centrale* strains [41], suggesting that it may not be *A. centrale*. Using registered *A. centrale* sequences, phylogenetic analyses using the Bayesian method revealed the possible divergence of the Japanese *A. centrale*. This was also confirmed using newly obtained partial *groESL* sequences (unpublished data). Based on the citrate synthase gene (*gltA*), *A. centrale* from Japan shares a similarity of only 74.5% to the only other registered *A. centrale* sequence (CP001759) from Israel (vaccine strain). Recently, *A. central* has been shown to be prevalent in wild sika deer in Shizuoka Prefecture, Japan [42].

### A. bovis

*A. bovis* often infects circulating monocytes [1,2] and tissue macrophages of cattle in Africa, Asia, South America, and some of the Caribbean Islands [42]. While *A. bovis* infections are rare [43], they have been reported in the Middle East, and Sri Lanka [1]. DNA fragments of this pathogen have also been detected in several animals in Japan including deer, raccoons, dogs, domestic cats, Hokkaido brown bear, cattle, and Japanese wildcats [8,10,39,40,44-50]. The role of these animals as a natural carrier of *A. bovis* needs to be clarified. On the other hand, the documented tick vector in Japan wherein *A. bovis* was molecularly detected is *Haemaphysalis megaspinosa* in cattle [15] and *H. longicornis* in deer and Japanese wildcats [39,41,51]. Recent phylogenetic analyses of *A. bovis* using 3 genes suggested that it is under the subclade of the genus where *A. phagocytophilum* and *A. platys* were found under the *Anaplasma* genus [14].

*A. bovis* infection is rare and may be subclinical in nature [10,11,23]. In clinical cases, it may be characterized by fluctuating fever lymphadenopathy, depression, and death. Principal manifestations include fever, anorexia, diarrhea, and rarely, central nervous system involvement [52]. Leukopenia and thrombocytopenia may occur [1]. Other possible signs include congestion of oral mucous membranes, decreased amount of stools, absent feces, constipation, dullness, swelling of head, face, ear, jaw and nasal area, lacrimation, mucoid nasal discharge, purulent nasal discharge, syncope, unthriftiness, and emaciation [42]. Some cattle found positive in Japan showed mild to severe anemia, mild leukocytosis and thrombocytopenia, but coinfections with *Anaplasma* sp. Japan and *Theileria* sp. are confounding factors which may have also caused the hematologic findings [11].

Diagnosis of *A. bovis* is usually by blood smear [8] or molecular methods [14]. Morulae are usually found in monocytes of infected cattle [53], however, no morulae in blood smears of *A. bovis* - positive cattle in Japan - were observed [11]. It may also be detected in blood films or organ smears, particularly lungs and liver [42]. Complete blood count can be performed as anemia and thrombocytopenia can also be seen [10]. On the other hand, molecular detection provides a reliable method in detecting *A. bovis*. Among which is the 16S rRNA gene-based nested polymerase chain reaction (nPCR) [39]. The same with *A. phagocytophilum*, thin slide films from liver, kidney, spleen, lungs, and peripheral blood should be prepared for microscopic examination at necropsy [54].

### A. phagocytophilum

The bacterium *A. phagocytophilum* is the recently designated name replacing three species of granulocytic bacteria, *Ehrlichia phagocytophila*, *Ehrlichia equi* and the agent of human granulocytic ehrlichiosis, now known as human granulocytic anaplasmosis (HGA) [55,56]. It is the agent of pasture fever or tick-borne fever of ruminants [57]. It is also known to infect humans and horses in the US, Europe [11] and some parts of the Middle East and Asia [58]. This pathogen is known to be highly adaptive to several vectors, hosts and seasonal variations [59]. The bacterium infects blood cells, although no studies have shown direct tropism to neutrophils in cattle as compared to humans [16]. HGA was first described in 1993. The potential threat of HGA to public health has been increasingly recognized in the United States and several European countries [43]. Ixodid ticks and *H. megaspinosa* can be important in the transmission of *A. phagocytophilum* in animals [13,15]. Ohashi *et al*. [60] suggested that *Haemaphysalis formosensis*, *H. longicornis*, and *Ixodes ovatus* may be associated with HGA in Japan.

DNA fragments of *A. phagocytophilum* were first reported in cattle on Yonaguni Island, Okinawa, Japan in 2006 [11]. It was later detected in cattle and sika deer in Hokkaido [10,11,39,61] and wild sika deer in Shizuoka, Japan [48]. Coinfection with *Borrelia* and *Rickettsia* spp. has also been reported in Thoroughbred horses in Hidaka district, Hokkaido [62]. Recently, 2 cases of HGA in 2013 [60] and additional 4 cases in 2014 [63] were confirmed in Japan. Infected patients showed fever, chills, headache, and malaise. *A. phagocytophilum* usually causes a disease in humans characterized by fever, headache, myalgia, leukopenia, anemia, and thrombocytopenia [54]. In ruminants, TBF primarily affects neutrophils and other granulocytes [23], which may develop to a severe febrile reaction, bacteremia and leukopenia due to neutropenia, lymphocytopenia, and thrombocytopenia within a week of exposure to a tick bite [64]. Reduced milk production in cattle can also be observed [65]. Growth rate can also be affected. Mortality is generally low but secondary infection can lead to death in some patients [23]. Subclinical infection has also been documented [66]. In Japan, infected animals can be asymptomatic [10], although a recent canine case showed clinical signs, including anorexia, fever, thrombocytopenia, neutropenia and high levels of liver enzyme activity and C-reactive protein [67]. Coinfection of *A. phagocytophilum* with other tick-borne pathogens may confound the observed clinical signs [49]. *A. phagocytophilum* can be detected in cattle without any sign of infection but is actually maintaining a persistent subclinical state. The severity of the infection may be influenced by several factors, including the variants involved, coinfection with other pathogens, age, immune status, host condition and environment (climate and management) [55,66].

In diagnosing *A. phagocytophilum* infection, clinical signs and blood smears are unreliable as the disease can be persistent and subclinical in humans and animals with no indications in the smears [55,66,68]. If visible, inclusion bodies may be seen in the neutrophils [69]. Blood in anticoagulant should be obtained for hematologic testing. In Giemsa-stained thin blood films, *Anaplasma* spp. appears as dense, homogeneously staining blue-purple inclusions 0.3-1.0 µm in diameter. However, it may be impossible to recognize infected blood cells by the traditional Giemsa staining method due to the low amount of infected cells. At necropsy, thin slide films from liver, kidney, spleen, lungs, and peripheral blood should also be prepared for microscopic examination [53].

Chronically infected carriers may be identified with a fair degree of accuracy by serologic testing. However, DNA-based detection methods are most useful in species and strain differentiation tests [64]. Serological examination by immunofluorescence assay and PCR was used in France to detect *A. phagocytophilum* in cattle [70]. Some also utilize competitive enzyme-linked immunosorbent assay using *Msp5* and monoclonal anti-*Msp5*, like the case in Sicily, Italy [71]. This assay is recommended by the International Office of Epizootics, otherwise known as Office International des Epizooties, for the serological diagnosis of bovine anaplasmosis, but was however shown to be cross-reactive among *A. marginale*, *A. centrale*, *A. ovis*, and *A. phagocytophilum* [72]. Kawahara *et al*. [39] stressed that the absence of cross protection among this *Anaplasma* spp. elucidate the potential difficulty in diagnosing *Anaplasma* spp. infections by serological tests and that molecular diagnosis of *Anaplasma* infection is more reliable. Ohashi *et al*. [60] suggested the use of propagation in THP-1 and HL60 cells for serodiagnosis to avoid misdiagnosis of infection in humans. Species-specific nPCR is also a reliable method in the molecular detection of the pathogen [10]. The 16S rRNA nPCR assay [39] is currently among the common methods of confirmation in Japan and other countries.

Different strains may infect animals and humans. High genetic diversity in the 16S rRNA and *Msp4* genes of *A. phagocytophilum* strains can be observed [73]. *A. phagocytophilum* strains can have different tropism, in which a strain from one animal may not cause disease in another species. Hence, with increasing reports of dissimilar genotypes from different regions of the globe, defining distinct phenotypes and using nomenclature that appropriately clarifies the distinctions are important [74]. It can be implicated that different variants may exist within the same herd, and even simultaneously within the same animal. Variants may behave differently and interact in the mammalian host [55].

### *Anaplasma* sp. Japan

In Japan, a potentially novel *Anaplasma* sp. closely related to *A. phagocytophilum* was detected [12]. It is also reported as “AP-sd” in wild brown bears and rodents in Hokkaido, Japan [75]. Several past studies have reported its detection as *A. phagocytophilum* despite low sequence similarities, including that of Wu *et al*. [48] where this species was detected in wild sika deer in Shizuoka, Japan using primers by Kawahara *et al*. [39] and classified it as *A. phagocytophilum* (deer strain). Detection of a potentially novel *Anaplasma* species detected from ticks and deer with diverse p44/msp2 gene sequences were also reported in several studies [57,76-78]. Based on the results of these studies, it may represent a new species because of its low sequences similarities with the citrate synthase (*gltA*), heat shock operon (*groESL*) and 16S rRNA genes, and the results using several methods of phylogenetic analysis [12,49,57,75]. To further validate the novelty, additional characterization using the ftsZ and p44/msp2 gene can be performed [75,79]. On the other hand, molecular methods have been developed to detect this species based on the *gltA* [13] and *grOESL* genes [14]. In addition, a reverse line blot hybridization based on the 16S rRNA gene was developed [61].

The genus *Anaplasma* can be pathogenic to humans and animals. Due to public health implications, continuous research in Japan is needed to monitor its epidemiology and distribution. Coinfection of *Anaplasma* spp. with other pathogens, including *Mycoplasma*, *Theileria* or *Babesia* spp., can also be possible due to common vectors and may have a clinical impact on the diseased animal [18,80-82]. Recently, *Anaplasma* DNA was detected in milk from goats and sheep [83] in Japan. It may be worthwhile to investigate its presence in the milk of dairy cattle in Japan because of its possible effects to production. On the other hand, further studies might be needed to establish the novelty and pathogenesis of the potentially novel *Anaplasma* species in Japan.

## Conclusion

There are several *Anaplasma* spp. that are of veterinary importance in Japan. The presence of these species must be monitored because of its potential impact to animal and human health.

## Authors’ Contributions

APY drafted the manuscript and HI collected the literatures and information about the related researches in Japan. Both authors contributed in incorporating their insights in the manuscript. Both authors read and approved the final manuscript.
